# Selective control of the contact and transport between droplet pairs by electrowetting-on-dielectric for droplet-array sandwiching technology

**DOI:** 10.1038/s41598-021-91219-x

**Published:** 2021-06-11

**Authors:** Satoshi Konishi, Chikara Ohya, Tatsuhiro Yamada

**Affiliations:** 1 Department of Mechanical Engineering, College of Science and Engineering, Ritsumeikan University, Kusatsu, 525-8577 Japan; 2 Graduate Course of Science and Engineering, Ritsumeikan University, Kusatsu, 525-8577 Japan; 3 Ritsumeikan Global Innovation Research Organization, Ritsumeikan University, Kusatsu, 525-8577 Japan

**Keywords:** Fluidics, High-throughput screening

## Abstract

Methodological advances in on-chip technology enable high-throughput drug screening, such as droplet-array sandwiching technology. Droplet-array sandwiching technology involves upper and lower substrates with a droplet-array designed for a one-step process. This technology is, however, limited to batch manipulation of the droplet-array. Here, we propose a method for selective control of individual droplets, which allows different conditions for individual droplet pairs. Electrowetting-on-dielectric (EWOD) technology is introduced to control the height of the droplets so that the contact between droplet-pairs can be individually controlled. Circular patterns 4 mm in diameter composed of electrodes for EWOD and hydrophilic–hydrophobic patterns for droplet formation 4 μl in volume were developed. We demonstrate the selective control of the droplet height by EWOD for an applied voltage up to 160 V and selective control of the contact and transport of substances. Presented results will provide useful method for advanced drug screening, including cell-based screening.

## Introduction

Lab-on-a-chip (LOC) and micro total analysis system (μTAS) technologies have had a strong impact on and provided great benefits to the biochemical field^1^. In the fields of LOC and μTAS, droplets are generated by various techniques. Droplet microfluidics technology, which differs from conventional microfluidics using continuous flow, has also been studied^2^. Droplet microfluidics in microchannels often involve emulsions such as an oil-in-water emulsion formed in the main flow by the interrupting branch flow. Droplets are generated in a microchannel using a T-junction in a popular method^3^. The main flow is divided into droplets by the interrupting branch flow at the T-junction where the side branch channel intersects the main channel.

Digital microfluidic technology involves droplets in open space on a substrate without limitation to the spaces in microchannels. A large number of droplets are arranged over the whole substrate plane. Distributed droplets are manipulated in parallel so that they are individually conveyed, fused together, and split into separate droplets. Electrical control of the wettability is regarded as a promising method for microfluidics; this method could provide a simple model of electrowetting-on-dielectric (EWOD) droplet actuation^4^. Washizu reported the electrostatic transport of droplets using distant electrodes^5^. Pollack et al. reported the transport of a droplet by EWOD^6^. Lee et al. reported pumping by electrowetting on a metal and EWOD^7^. Cho et al. reported digital microfluidic circuits obtained by electrowetting^8^. Droplets can be electrically manipulated using electrodes on the substrate^9^. In a digital microfluidics, droplets are generated from a reservoir by EWOD^8^. In addition, the wettability control on the substrate surface can be used to generate droplets. Patterns defined by hydrophilic/hydrophobic surfaces can be formed. One can use various materials and structures to provide hydrophilic/hydrophobic surfaces. Polydimethyl siloxane, polytetrafluoroethylene, and Cytop^®^ are regarded as typical hydrophobic materials for μTAS. A self-assembled monolayer (SAM) is used for surface modification as the hydrophobic material. Polyvinyl alcohol is often used to modify a hydrophobic surface into a hydrophilic surface. O_2_ plasma treatment is also used to enhance the hydrophilicity, whereas CF_4_ plasma improves the hydrophobicity. SiOx and SiCx, which are types of silicon compounds, provide hydrophilic and hydrophobic surfaces, respectively. TiO_2_ is well known as a material whose wettability can be changed by the photocatalyst effect^10–13^. In addition to the material dependence of the wettability, the surface morphology, such as surface roughness, also affects the wettability of a surface. The Wenzel model and Cassie–Baxter model have been used to explain the mechanism^14,15^.

Droplets are formed along the stream in the microchannels. Digital microfluidics involves the open and in-plane space on a substrate. Conventional in-plane digital microfluidic systems are open systems, so they are accessible for the introduction and sampling of liquid and samples by pipetting. Levkin et al. reported a droplet-array sandwich chip^16,17^ that was developed for cell-based high-throughput screening. This droplet-array sandwiching technology used thousands of microdroplets containing cells on a glass slide with a preprinted library and a superhydrophobic–superhydrophilic pattern. The droplet-array sandwich chip was designed for one-step cell seeding and simultaneous initiation of screening. Subsequently, the same group reported the parallel single step addition of different chemicals into microdroplets^18^ as well as biochemical application^19^.

A three-dimensional cell culture for a cellular aggregate, such as a spheroid, is important for providing cellular models for biological research and pharmaceutical science. A hanging-drop culture plate was reported for high-throughput 3D spheroid culture and drug testing^20,21^. Hanging-droplet technology allows three-dimensional cell culture on a substrate positioned upside down to generate hanging droplets containing cells^22,23^. Droplet-array sandwiching technology allows droplets on one substrate to have face-to-face access to the corresponding droplets on the other substrate. Zhang et al. reported high-throughput superhydrophobic microwell arrays for investigating multifactorial stem cell niches^24^. They developed a novel superhydrophobic microwell array chip featuring physical separation of each microwell by a grafted layer of superhydrophobic polymers. Medium exchange could be completed by simply submerging the entire chip.

**Figure 1:**
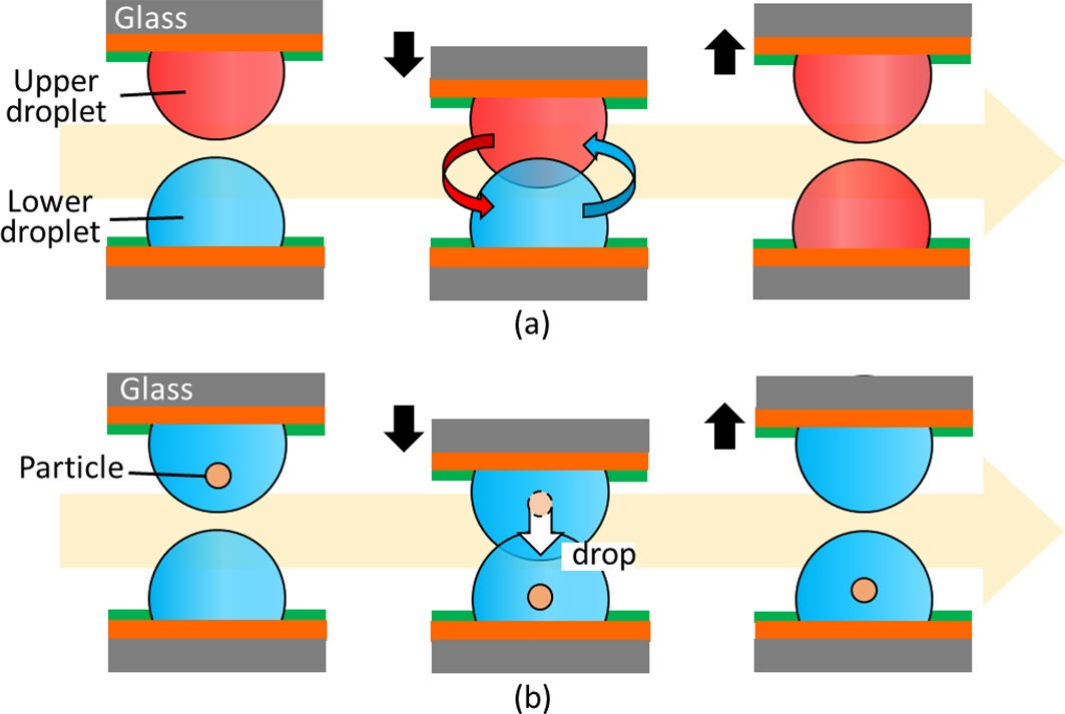
Contact and transport between droplet pairs by droplet-array sandwiching technology. One droplet pair is depicted for explanation. Droplets are generated and prepared on different substrates with hydrophilic–hydrophobic patterns. The two substrates are arranged facing each other by aligning corresponding droplet pairs in the vertical direction. The corresponding droplets come into contact and fuse when opposing substrates are moved closer together. (**a**) Illustration of a mixture of two droplets for concentration control of substances. Substances in the upper droplet diffuse into the lower droplet through the fused contact part and mix in the fused droplet pair. (**b**) Illustration of particle transport between droplet pairs. Particles in the upper droplet move into the lower droplet.

**Figure 2:**
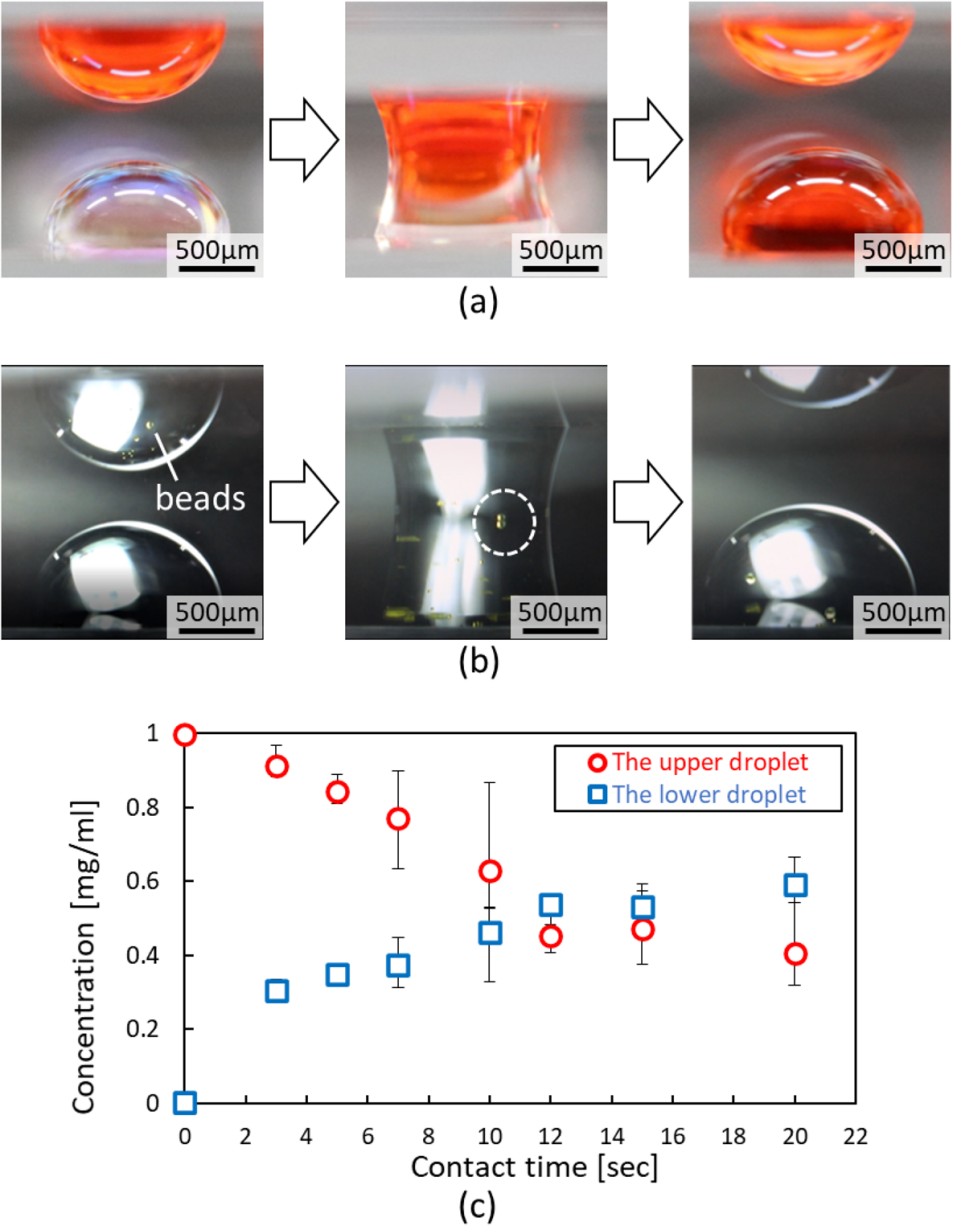
More detailed analysis of the mixture and transport in addition to our previous report^25^. The hydrophilic–hydrophobic patterns for droplet formation are prepared by the combination of TiO_2_ and an octadecylphosphonic acid self-assembled monolayer (ODP-SAM). The volume of a droplet formed on the circular pattern 2.48 mm in diameter is 4 μl with an assumed contact angle of 90°. (**a**) Mixture by the contact fusion of an upper droplet of the red dye compound new coccine and a lower droplet of deionized (DI). Three sequential photographs show a droplet pair before contact, fused droplets, and a droplet pair after separation from the left. (**b**) Transport of particles between a droplet pair. Fluorescence beads moved from an upper droplet to a lower droplet through a fused contact. (**c**) Detailed evaluation of the mixture between the droplet pair in (**a**). The change in the concentration of substance in the droplet pair is further examined in accordance with the contact time. The concentration change of the red dye compound new coccine is evaluated for a longer time than in our previous report^25^. The concentrations of new coccine in the upper and lower droplets appear to asymptotically converge to the intermediate value, approximately 0.5 mg/ml. The concentrations of the upper and lower droplets continue to decrease and increase at a moderate rate, respectively, after the concentrations intersect at the intermediate value.

One of the authors also reported the spatial contact of droplets in a manner similar to droplet-array sandwiching technology^25^. As mentioned above, in droplet-array sandwiching technology, another opposing upper substrate is introduced as a substitute for pipettes above the lower substrate^16–18^. In contrast to conventional droplet manipulation within a two-dimensional space, droplet-array sandwiching technology expands the available space to three dimensions by introducing the upper substrate. The samples in the droplets or the droplets themselves can be exchanged between the lower and upper substrates by using the droplet-array sandwiching technology in addition to the conventional in-plane manipulation of droplets on the individual substrates. Figure 1a illustrates the mixture of two droplets for concentration control of substances. Figure 1b depicts particle transport between the droplets as a representative manipulation of droplets. Figure 2a,b demonstrate the mixture and transport by droplet-array sandwiching technology^25^, where hydrophilic–hydrophobic patterns were formed by the combination of TiO_2_ and an octadecylphosphonic acid self-assembled monolayer (ODP-SAM). Figure 2c presents the change in the concentration of substance in each pair of droplets in accordance with the contact time.

Figure 2a, corresponding to Fig. 1a, reports the mixture of two droplets for concentration control of substances. Here, the upper and lower substrates were regarded as the supplier and receiver substrates, respectively. Figure 2a shows the contact fusion of a droplet of the red dye compound new coccine on the upper supplier substrate and a deionized (DI) water droplet on the lower receiver substrate. A water solution of 1 mg/ml new coccine was prepared. Each droplet was designed to be 4 µl in volume. The upper and lower droplets came into contact and fused when they were moved closer together in the vertical direction. A mixture of liquid as the solvent and diffusion of the substances occurred between the fused droplets. After sufficient time passed, the fused droplets were separated. The mixture and diffusion of new coccine between the upper droplet and lower droplet in a pair was evaluated as a result of a more detailed analysis in addition to a previous report, as shown in Fig. 2c^25^. The details of this result in Fig. 2c are discussed and explained later. The transport of particles by gravity from the upper droplet to the lower droplet was attempted (Fig. 2b). Fluorescent beads of 90 μm diameter were successfully transported between the upper and lower droplets using the contact fusion of droplets.

Further attempt was made to apply the spatial contact of droplets to biological applications^25^. Droplets are regarded as miniaturized chambers for various biochemical reactions. Hanging droplets on an inverted substrate were used for the culture of cells such as spheroids^21–24^. In our work^25^, a Madin–Darby canine kidney (MDCK) cyst was used as a living cellular aggregate of approximately 100 μm diameter. The living cellular aggregate was successfully transported to the lower droplet within 10 s. Sequentially, medium exchange was accomplished by using spatial contact fusion of droplets every day. Cell viability was confirmed three days after the administration of calcein AM.

This work proposes EWOD technology for the selective control of contact between a pair of droplets through the control of the height of droplets in droplet-array sandwiching technology. The authors presented preliminary results using EWOD^26^. We could control the height of the droplet by EWOD, where the droplet was formed on parylene C covering electrodes for EWOD. Previous work could not restore the droplet in the initial height after the voltage for EWOD was turned off. This work has improved droplet formation on the electrodes by patterning hydrophobic and hydrophilic materials on a substrate. The wettability pattern for droplet formation contributes to form a droplet at a designed position for the shape control by EWOD. This paper presents further possibilities for droplet-array sandwiching technology by relaxing the restriction on batch manipulation of droplet arrays. We anticipate that our technology will be a useful substitute for conventional multiple pipetting beyond the current droplet-array sandwiching technology limited to batch operation.

## Results and discussion

Droplet-array sandwiching technology allows contact of droplets and transport between droplet pairs. Figure 2c examines the mixture and diffusion of new coccine which is a red dye as a typical substance. A water solution of 1 mg/ml new coccine was prepared. Each droplet was designed to be 4 µl in volume. The upper and lower droplets came into contact and fused when they were moved closer together in the vertical direction. A mixture of liquid as the solvent and diffusion of the substances occurred between the fused droplets. After sufficient time passed, the fused droplets were separated. The concentration of new coccine in the upper and lower droplets appeared to asymptotically converge to an intermediate value, approximately 0.5 mg/ml. The concentrations of the upper and lower droplets continued to decrease and increase at a lower rate, respectively, after the concentration intersected at the intermediate value.

### Design of wettability patterns with electrodes for EWOD

**Figure 3:**
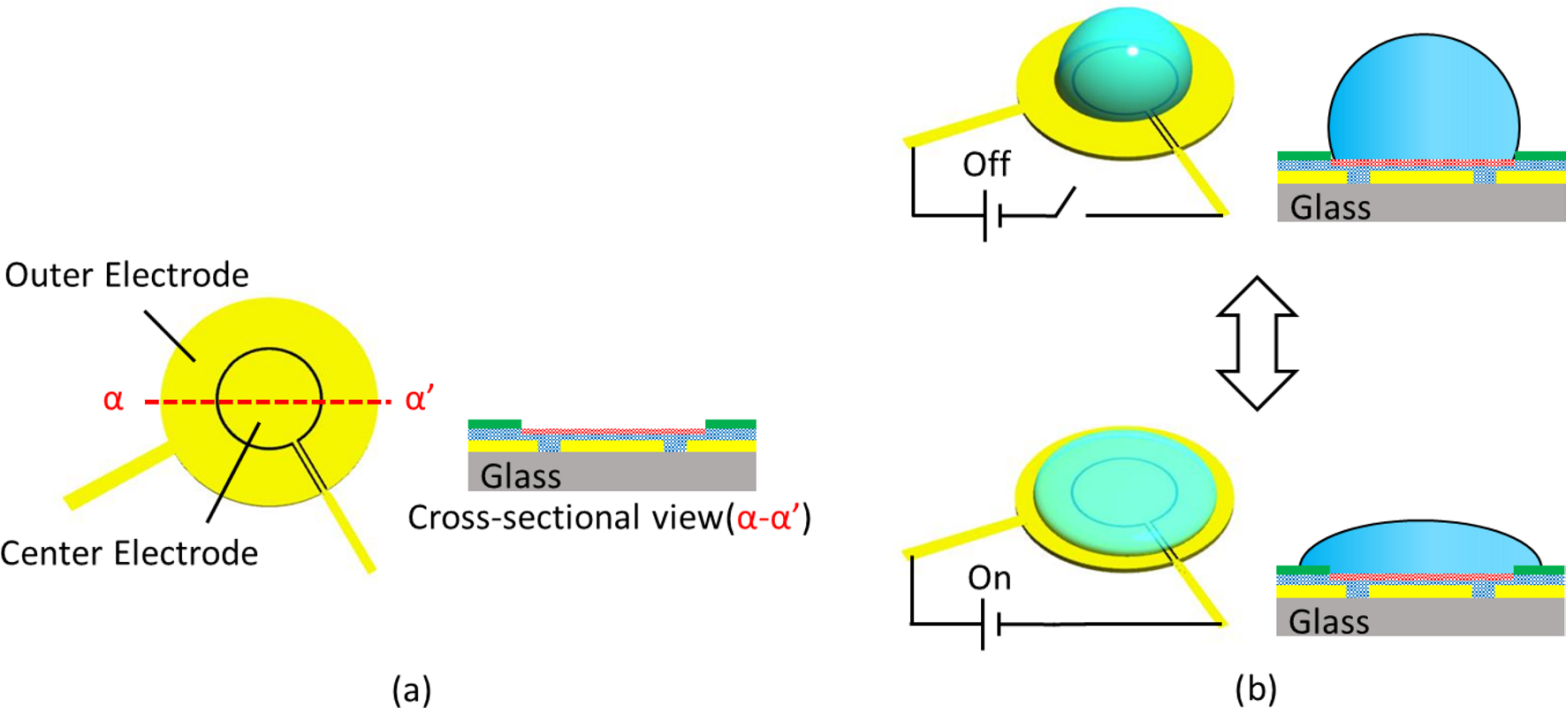
Height control of droplet by EWOD technology. (**a**) Electrode design for EWOD. The center electrode and outer electrode, which are insulated from each other, are designed in a circular pattern for droplet generation. (**b**) An electric field for EWOD is generated by the applied voltage between two electrodes. The contact angle of the droplet decreases with the electric field for EWOD when a voltage is applied. The outer electrode is set at high voltage potential. Due to the change in contact angle, the height of the droplet changes from its initial height [top drawing in (**b**)] to a lower height [bottom drawing in (**b**)].

Selective contact control technology by EWOD allows the transport of substances between selected droplet pairs in a droplet-array. The height of a droplet is controlled by EWOD technology, as shown in Fig. 3. Preliminary results were reported in^26^. The contact angle on the high potential electrode decreases when a voltage is applied^27,28^. Each elemental circular electrode is composed of center and outer electrodes, where a droplet is prepared as shown in Fig. 3^26^. Therefore, a voltage is applied so that the outer electrode has a higher potential than the center electrode. Then, the droplet changes shape, and the droplet height decreases. Contact/noncontact between a pair of droplets can be selected by controlling the height of the droplets. Selective contact control technology allows the transport of substances between selected droplet pairs in droplet arrays, whereas the transport of substances does not occur between noncontacting droplet pairs. This paper presents EWOD technology for selective contact control in droplet-array sandwiching technology.

### Fabrication results of wettability patterns with electrodes for EWOD and droplet generation

**Figure 4:**
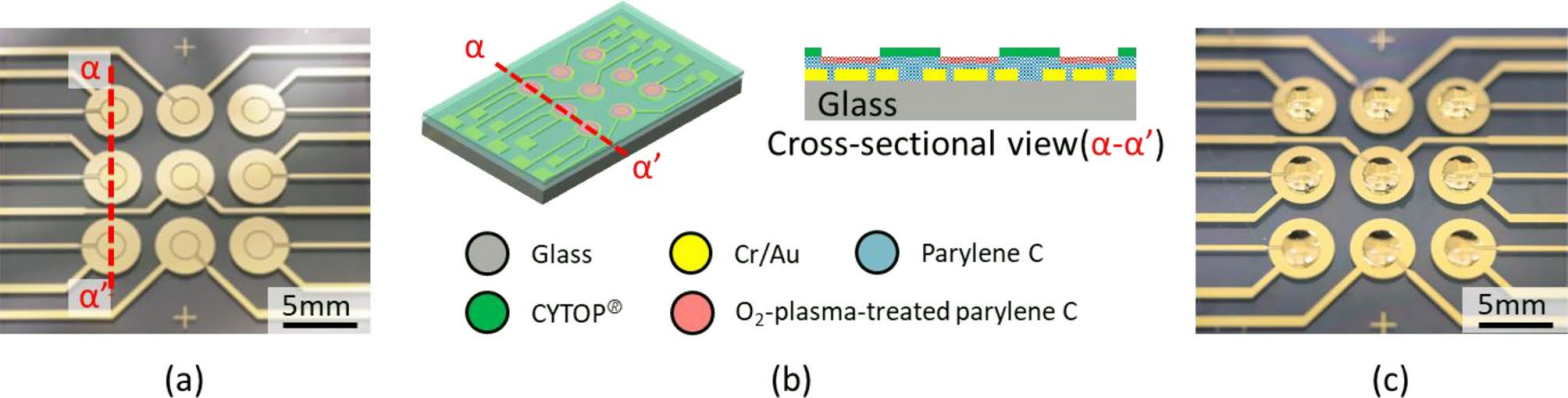
Fabrication result of the device for height control of droplets by EWOD technology. (**a**) Top view of fabricated device with three by three arrays of circular patterns. Each circular pattern for a droplet has center and outer electrodes under the hydrophilic–hydrophobic pattern. The diameters of the hydrophilic–hydrophobic pattern and outer electrode are 2.48 mm and 4 mm, respectively. An initial hemispherical droplet on the 2.48 mm diameter circular pattern of the hydrophilic–hydrophobic surface has a 4 μl volume with a 90° contact angle. The diameter of the center electrode is 1.86 mm, and the gap between the center and outer electrodes is 70 µm. (**b**) Schematic and cross-sectional drawings of the device. A cross-section of red dotted lines in (**a**) and a schematic drawing are shown. Cr (70 nm thick)/Au (200 nm thick) electrodes are patterned on a glass substrate and coated and insulated by 1 μm-thick-parylene C. The parylene C is partially treated by O_2_ plasma to obtain a hydrophilic surface. 12 nm-thick-Cytop^®^, a hydrophobic material, is patterned to define the circular pattern of hydrophilic parylene C treated by O_2_ plasma for droplet generation. (**c**) Three by three arrays of DI water droplets are prepared on the device.

Figure 4a shows a top view of the fabricated device, with three by three arrays of circular patterns. Electrodes composed of center and outer electrodes for height control of droplets by EWOD were fabricated on the substrate, where hydrophilic–hydrophobic patterns were fabricated above the electrodes. A droplet was prepared on a circular pattern of a hydrophilic–hydrophobic layer by a combination of O_2_-plasma-treated parylene C and Cytop^®^. Parylene C was employed for dielectric material for EWOD because of its dielectric characteristics and smooth surface coverage over the electrodes. A pattern of Cytop^®^ on palylene C provides a stable hydrophilic–hydrophobic pattern for droplet formation, whereas our previous report used only palylene C layer over the electrodes^26^. Cytop^®^ in combination with parylene C was selected to avoid damage of parylene C by sputtering deposition of TiO_2_ as hydrophilic surface. Parylene C is suitable for dielectric layer for EWOD, and, what is better, wettability of parylene C can be treated to hydrophilic characteristics by O_2_ plasma. Therefore, the combination of O_2_-plasma-treated parylene C and Cytop^®^ was designed in this study. The diameters of the hydrophilic–hydrophobic pattern and outer electrode were 2.48 mm and 4 mm, respectively. An initial hemispherical droplet on the 2.48 mm diameter circular pattern of the hydrophilic–hydrophobic surface had a 4 μl volume with an assumed contact angle of 90°. The diameter of the center electrode was 1.86 mm, and the gap between the center and outer electrodes was designed at 70 µm for electric field generation. The electrodes were connected to a peripheral voltage controller including a DC power supply. Figure 4b depicts schematic and cross-sectional drawings of the device. A cross-section of red dotted lines of Fig. 4a and schematic drawing was shown. Cr (70 nm thick)/Au (200 nm thick) electrodes were patterned on a glass substrate and coated and insulated with parylene C. The parylene C was partially treated by O_2_ plasma to obtain hydrophilic surface. Cytop^®^, a hydrophobic material, was used to define the circular pattern of the hydrophilic parylene C treated by O_2_ plasma for droplet generation. It is important to decrease the distance between the electrodes and droplets to apply an effective electric field for EWOD. The distance between the electrodes and droplets was determined by the thickness of the insulating layer and hydrophilic–hydrophobic layer. The parylene C layer worked as both insulating and hydrophilic material. The thickness of parylene C was designed to be 1 μm in consideration of dielectric strength, whereas the thickness of Cytop^®^ was prepared at 12 nm to minimize the distance between electrodes and droplets. Figure 4c shows three by three arrays of DI water droplets prepared on the device.

The contact angle θ was measured by the θ/2 method. The contact angle was determined by using the radius and height of the droplet. A droplet was formed on a circular pattern composed of O_2_-plasma-treated parylene C and Cytop^®^. The contact angle on the parylene C was 92.2° and changed to 14.3° after surface treatment by O_2_ plasma, whereas the contact angle on the Cytop^®^ in the outer region of the O_2_-plasma-treated parylene C remained constant at 110.9°.

### Height control of a droplet by EWOD for selective contact control

**Figure 5:**
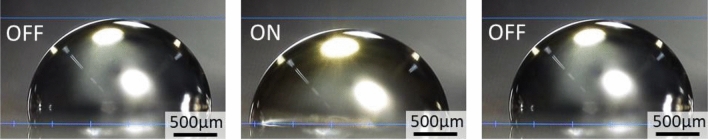
Experimental results of height control of a droplet by electric field for EWOD. A 4 µl DI water droplet is prepared on a circular pattern for a droplet. Side views of the droplet are observed as the applied voltage is turned off and on. The height of the DI water droplet is successfully decreased through the application of an electric field. The height of the droplet, which is 1290 μm in the initial state, decreases to 1101 μm when a voltage of 160 V is applied, as shown in this figure. The height of the droplet increases when the applied voltage is turned off.

The shape of a droplet can be changed by an electric field with EWOD due to the change in the surface tension on the substrate. The device with three by three arrays of electrodes shown in Fig. 4 was used to demonstrate height control of droplets by EWOD. Figure 5 and Supplemental Video S1 show the experimental results of height control of a droplet. A 4 µl DI water droplet was prepared on a circular pattern for a droplet where a hydrophilic–hydrophobic pattern was fabricated above the center and outer electrodes. Side views of the droplet at 0 V/160 V are compared in Fig. 5. The height of the DI water droplet was successfully decreased by applying an electric field. The height of the droplet was 1290 μm in the initial state without an applied voltage. The height of the droplet decreased to 1101 μm when a voltage of 160 V was applied for EWOD. The height of the droplet increased when the applied voltage was turned off. The hysteresis in height change of a droplet was observed when the voltage for EWOD was controlled. The selective control of the contact between droplet pairs is, however, not directly affected by the hysteresis because it uses the highest point of droplet without EWOD and the lowest point with EWOD. The repeated characteristics of height control are further examined and discussed later with Fig. 7.

**Figure 6:**
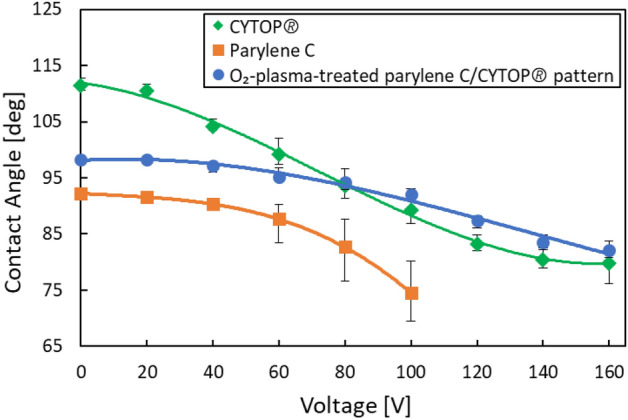
Material dependence of the contact angle control of droplets by an electric field. We form 4 μl droplet with an initial height of 1293 µm on a circular pattern with electrodes. A voltage is applied between the center and outer electrodes for electric field generation. The droplet changes shape, and the droplet height decreases. The contact angles of the droplet for an applied voltage of 0–160 V are measured. The contact angle decreases with increasing voltage. The phenomena on uniform surfaces of parylene C and Cytop^®^ are compared with the circular pattern composed of O_2_-plasma-treated parylene C and Cytop^®^. The contact angles on all surfaces decrease when a voltage is applied. In the initial state without voltage for EWOD, the hydrophobic Cytop^®^ surface shows a higher contact angle than parylene C, whereas the circular pattern composed of O_2_-plasma-treated parylene C shows an intermediate angle between the others. The contact angles of Cytop^®^ and that in the circular pattern are similar at high voltage.

We investigated the height change of a droplet by EWOD with the aim of controllability improvement. In general, the contact angle on the high potential electrode decreased when a voltage was applied. A droplet of 4 μl volume with an initial height of 1293 µm was formed on the electrode. A voltage was applied between the center and outer electrodes for electric field generation, where the outer electrode had a higher potential than the center electrode. The contact angles of the droplet for an applied voltage of 0–160 V are evaluated in Fig. 6. Contact angles in Fig. 6 were individually measured at each voltage. Figure 6 also compares uniform surfaces of parylene C and Cytop^®^ with the circular pattern composed of O_2_-plasma-treated parylene C and Cytop^®^. The contact angles on all surfaces decreased when a voltage was applied. The droplet height decreased due to the decrease in contact angle in accordance with increasing voltage.

**Figure 7:**
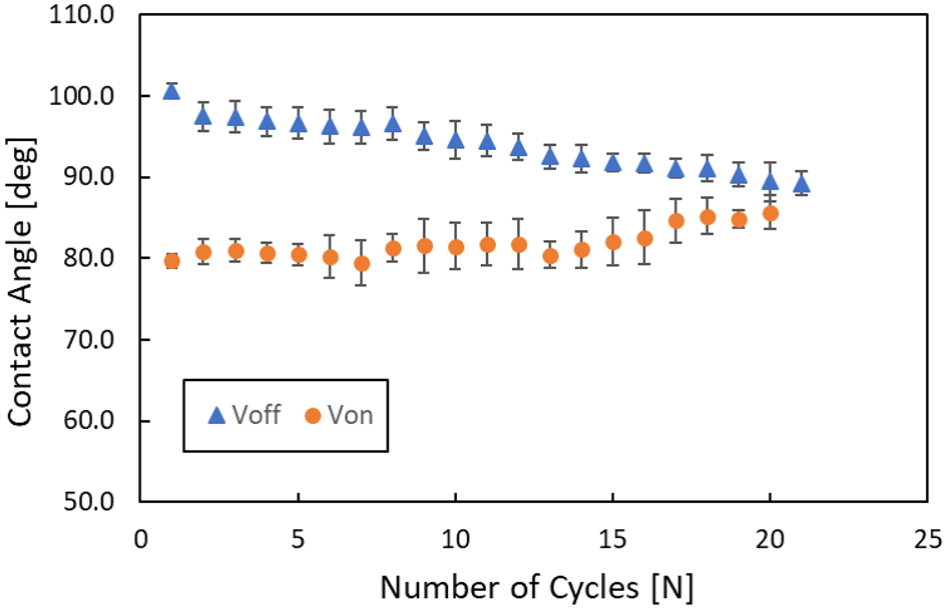
Repeatability of the height control of a droplet by EWOD. The wettability of the circular pattern composed of O_2_-plasma-treated parylene C and Cytop^®^ is estimated through repeated tests of 20 cycles. The contact angle changes after the first operation by EWOD and maintains a similar angle for a while after the first change. The change in the contact angle becomes nonnegligible between 5 and 10 cycles. When the switching of EWOD is repeated for 20 cycles, the difference between the contact angle with/without EWOD becomes too small to control the contact of the droplet pairs.

Figure 7 shows the repeatability in the height control of a droplet by EWOD. The contact angle of the circular pattern composed of O_2_-plasma-treated parylene C and Cytop^®^ was estimated through a repeated test of 20 cycles. The initial contact angle with/without voltage for EWOD was 79.7°/100.7°. The contact angle degraded approximately 3° after the first operation of EWOD and maintained a similar angle for a while after the first change. The change in the contact angle became nonnegligible between 5 and 10 cycles. When the switching of the EWOD was repeated for 20 cycles, the difference between the contact angle with/without EWOD became too small to control the contact of droplet pairs. These results have good agreement with previous report on degradation of electrowetting effect by repeated operation^29^. Meanwhile, electrowetting on liquid-infused film (EWOLF) was reported as a solution for reversibility of droplet shape^30,31^. These reversible electrowetting technology has potential to improve the drawback of conventional EWOD.

### Selective control of contact and transport for droplet-array sandwiching technology

**Figure 8:**
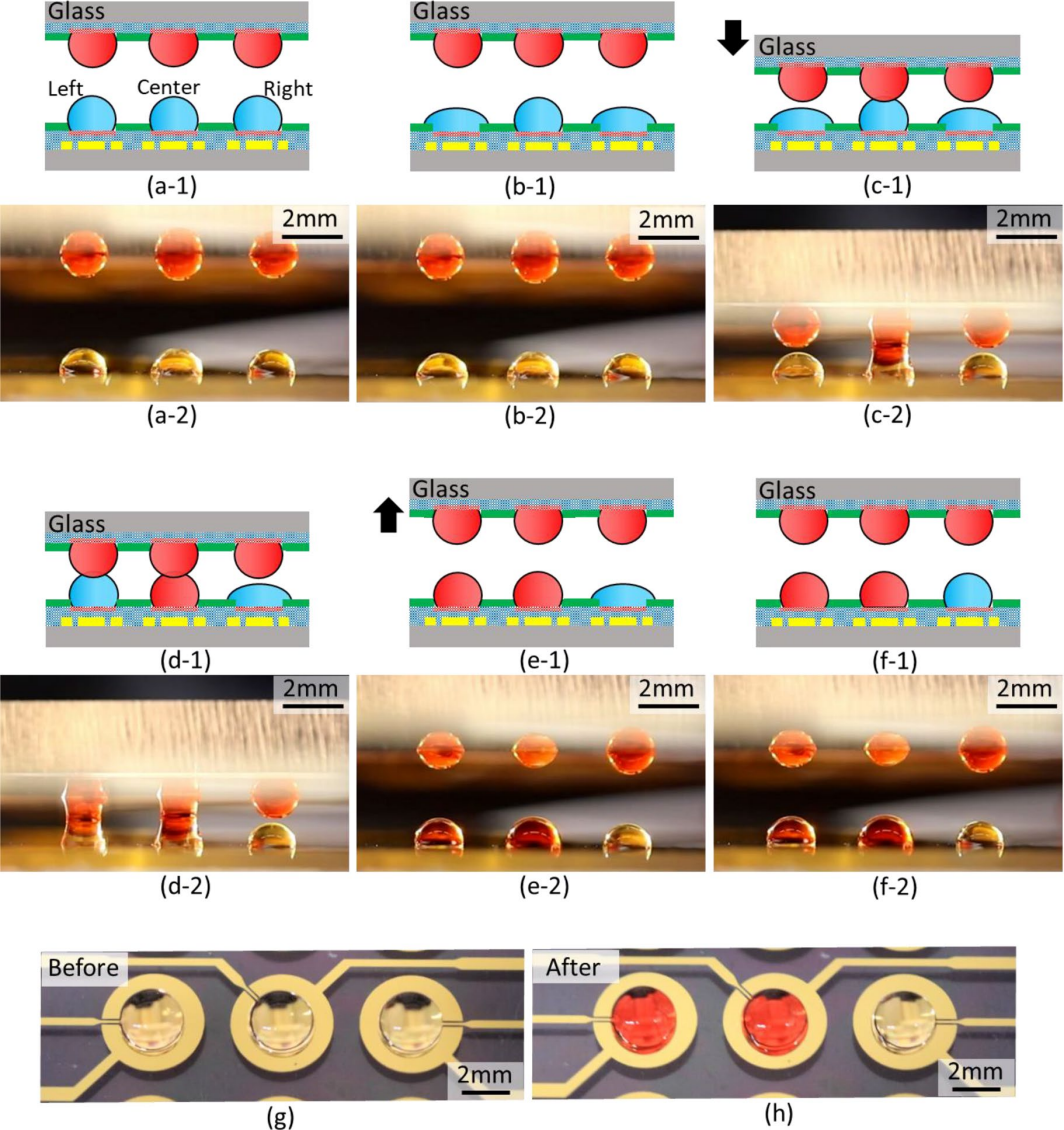
Demonstration of selective control of contact and transport for droplet-array sandwiching technology. (**a**) Three droplets of the red dye compound new coccine are prepared on the upper device whereas three droplets of DI water are prepared on the lower device. The volume of each droplet is assumed to be 4 μl. (**b**) Two droplets at both ends on the lower device are lowered by applying voltage whereas the central droplet maintains its initial height. (**c**) The position of the upper device is lowered to move individual droplet pairs closer together in the vertical direction. The droplet pairs at both ends do not contact yet when the central droplet pairs come into contact and fuse. New coccine is transported between the central droplet pairs. (**d**) The height of a droplet at the left end on the lower substrate is reverted by turning the applied voltage off. The droplet pairs at the left end come into contact and fuse. New coccine is transported between the left droplet pairs. (**e**) The position of the upper device is raised to separate the contact of droplet pairs. (**f**) The applied voltage to the droplet at the right end on the lower substrate is then turned off. Contact and transport do not occur between the droplet pairs at the right end. (**g**) Top view of an initial state of droplets on the lower device in (**a**). (**h**) Top view of a final state of droplets on the lower device in (**f**). Selective control of contact of the droplet pairs and transport of substances by EWOD are successfully demonstrated.

The height of a droplet could be changed by an electric field for EWOD. Next, selective control of contact and transport for droplet-array sandwiching technology was demonstrated by using three pairs of droplets as shown in Fig. 8 and Supplemental Video S2. Figure 8 shows still images from Supplemental Video S2. Three droplets of the red dye compound new coccine were prepared on an upper device whereas three droplets of DI water were prepared on a lower device (see Fig. 8a). The volume of each droplet was assumed to be 4 μl. Two droplets at both ends on the lower device were lowered by applying voltage whereas the central droplet remained at its initial height (see Fig. 8b). The position of the upper device was lowered to move individual droplet pairs closer together in the vertical direction (see Fig. 8c). The droplet pairs at both ends did not contact yet when the central droplet pairs came into contact and fused. New coccine was transported between the central droplet pairs. The height of a droplet at the left end on the lower substrate was reverted by turning the applied voltage off. The droplet pairs at the left end came into contact and fused (see Fig. 8d). New coccine was transported between the left droplet pairs. The position of the upper device was raised to separate the contact of droplet pairs (see Fig. 8e). The voltage applied to the droplet at the right end on the lower substrate was then turned off. Contact and transport did not occur between the droplet pairs at the right end. Selective control of the contact of droplet pairs and transport of substances by EWOD were successfully demonstrated.

## Materials and methods

### Fabrication of hydrophilic–hydrophobic patterns and materials

**Figure 9:**
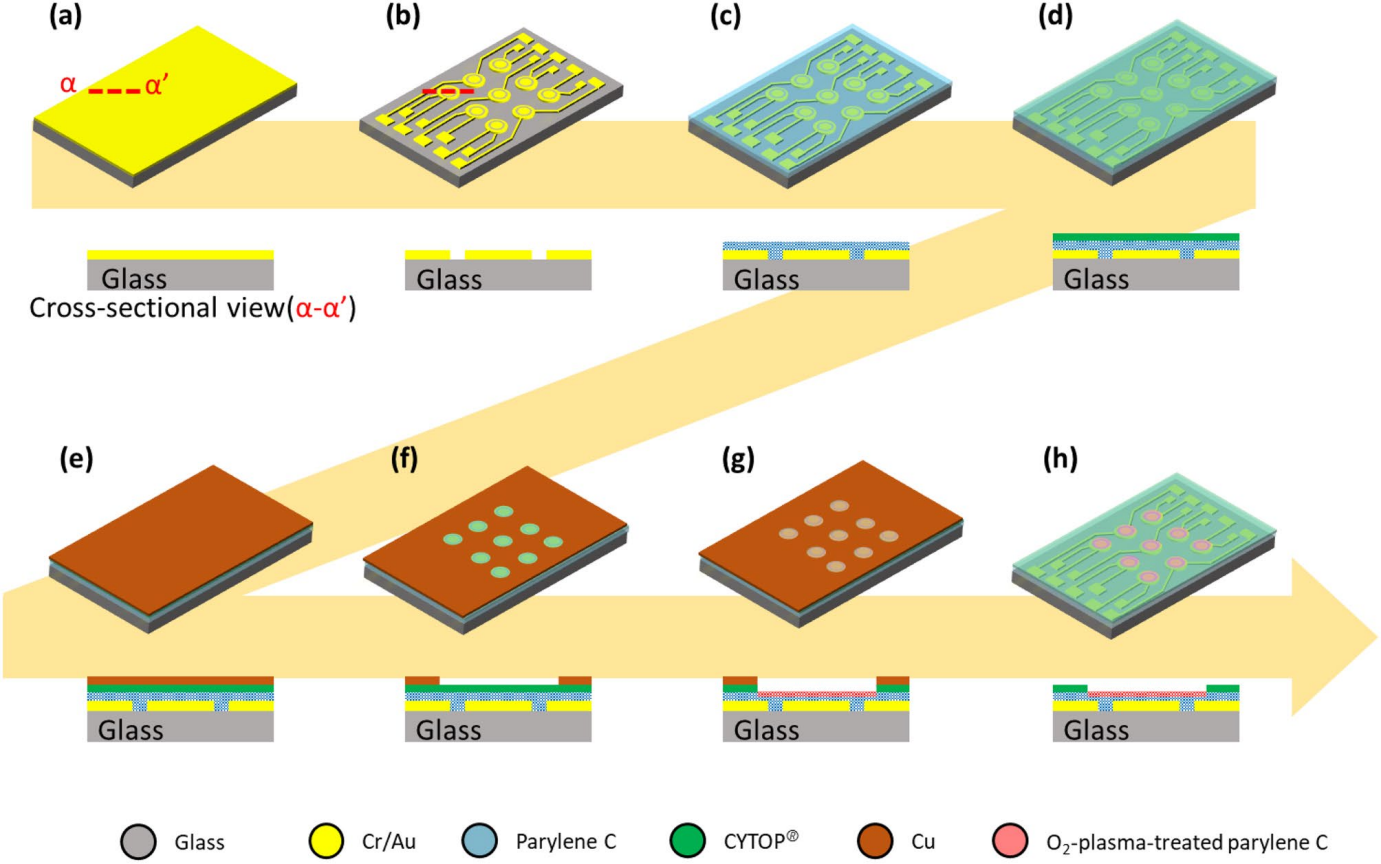
Fabrication process of hydrophilic–hydrophobic patterns above electrodes for EWOD. Cross-sectional drawings along the red dotted lines are also presented. (**a**) Deposition of Cr (70 nm thick) and Au (200 nm thick) on a glass substrate. (**b**) Patterning of circular patterns of elemental electrodes composed of center and outer electrodes. (**c**) Deposition of 1 μm thick parylene C as an insulating layer on the substrate. (**d**) Coating of a 12 nm thick CYTOP^®^ layer as a hydrophobic layer. (**e**) Evaporation of the Cu layer on the CYTOP^®^ layer. (**f**) Patterning of Cu for opening of circular patterns of CYTOP^®^. (**g**) O_2_-plasma-etching of CYTOP^®^ followed by O_2_-plasma-treatment of exposed parylene C under CYTOP^®^. (**h**) Removal of Cu mask followed by annealing at 250 °C for 180 min.

In this study, electrodes were prepared on a substrate for the application of an electric field to induce a height change in a droplet. In parallel, a hydrophilic area surrounded by hydrophobic material was used to generate a droplet on the substrate. The hydrophilic–hydrophobic patterns were micromachined in batches. Figure 4 illustrates the hydrophilic–hydrophobic pattern on the substrate. Figure 9 depicts the fabrication processes of the electrodes for EWOD and the hydrophilic–hydrophobic patterns. Cr (70 nm thick) and Au (200 nm thick) were deposited and patterned on a glass substrate to form the electrode (Fig. 9a,b). Each elemental circular electrode (4 mm in diameter) was composed of center and outer electrodes. The gap between the center and outer electrodes was designed to be 70 µm for electric field generation. Then, 1 µm thick parylene C was deposited as an insulating layer on the substrate (Fig. 9c). After completion of the fabrication process, parylene C works as a hydrophilic material as well. A 12 nm thick CYTOP^®^ layer was coated as a hydrophobic layer (Fig. 9d). Cu was evaporated and deposited on the CYTOP^®^ layer (Fig. 9e). Then, Cu was patterned to open and expose circular patterns of CYTOP^®^ (Fig. 9f). Exposed CYTOP^®^ was etched by O_2_ plasma to reveal parylene C surface. The parylene C surface was treated with O_2_ plasma to improve the wettability (Fig. 9g). O_2_ plasma treatment was applied for 180 s under the condition with the power of 50 W and O_2_ gas flow rate of 30 sccm. The Cu mask was removed and followed by annealing at 250 °C for 180 min (Fig. 9h). Annealing with the Cu layer was effective for the stability of the hydrophobic characteristics of the CYTOP^®^ layer.

### Materials for fundamental evaluation

The mixture of liquid as the solvent and the diffusion of the substances were evaluated in the fundamental experiments. New coccine (FUJIFILM Wako Pure Chemical Corporation, Japan) was used as a red dye compound for coloring. A Nano Drop One (Thermo Fisher Scientific Inc.) was used to measure the concentration of new coccine. Droplet pairs were contacted first and separated after the appointed time. The droplets separated at each appointed time were recovered, and their concentrations of new coccine were evaluated. 2 μl is extracted from individual droplets and analyzed their concentration of new coccine by an absorbance measurement.

Fluorescent beads (90 μm in diameter, Fluoresbrite Plain Microspheres, Polysciences, Inc.) were used to estimate the transport of particles between the fused droplets and remote droplets.

### Measurement method of contact angle of a droplet

Contact angle θ of the formed droplet was calculated by the θ/2 method or half-angle method. The θ/2 method uses a line drawn from the triphase point to the apex of the droplet assuming that the droplet shape is one part of the circle. The angle θ_1_ for the solid surface of the straight line connecting the endpoint to the top of the droplet is calculated by measuring the radius r and height h of the circle. The contact angle θ is calculated by doubling θ_1_.

### Setup for height positioning and voltage supply for EWOD

The upper substrate for the droplet-array sandwiching technology was lowered and raised by a precise positioning setup. The positioning setup was composed of a motorized horizonal Z-axis stage (KHE04006-C SURUGA SEIKI Co., Ltd.) and X–Y stage (KXL06100-N2-F SURUGA SEIKI Co., Ltd.) whose positioning accuracy were 2 µm and 4 µm, respectively. A digital microscope (VHX 500F, VH-Z50L, Keyence Corporation) was used to observe the device and droplet from the side. The horizontal alignment was accomplished in combination of the observation by the digital microscope and the X–Y stage. A DC power supply (DC160-7.2, NF Corporation) was used to supply voltage for EWOD.

## Supplementary Information

Supplementary Video S1.

Supplementary Video S2.

## Data Availability

All data generated or analyzed during this study are included in this published article.

## References

[1] Mark D, Haeberle S, Roth G, Stetten F, Zengerle R (2010). Microfluidic lab-on-a-chip platforms: Requirements, characteristics and applications. Chem. Soc. Rev..

[2] Teh SY, Lin R, Hung LH, Lee AP (2008). Droplet microfluidics. Lab Chip.

[3] Xu JH, Li SW, Tan J, Luo GS (2008). Correlations of droplet formation in T-junction microfluidic devices: From squeezing to dripping. Microfluid. Nanofluid..

[4] Nelso WC, Kim CJ (2012). Droplet actuation by electrowetting-dielectric (EWOD): A review. J. Adhes. Sci. Technol..

[5] Washizu M (1998). Electrostatic actuation of liquid droplets for micro-reactor applications. IEEE Trans. Ind. Appl..

[6] Pollack MG, Fair RB, Shenderov AD (2000). Electrowetting based actuation of liquid droplets for microfluidic applications. Appl. Phys. Lett..

[7] Lee J, Moon H, Fowler J, Schoelhammer T, Kim CJ (2002). Electrowetting and electrowetting-on-dielectric for microscale liquid handling. Sensor Actuators A.

[8] Cho SK, Moon H, Kim CJ (2000). Creating, transporting, cutting, and merging liquid droplets by electrowetting-based actuation for digital microfluidic circuits. IEEE J. Miecroelectromech. Syst..

[9] Yi UC, Kim CJ (2006). Characterization of electrowetting actuation on addressable single-side coplanar electrodes. J. Micromech. Microeng.

[10] Zhang X, Jin M, Liu Z, Fujishima A (2007). Superhydrophobic TiO_2_ surfaces: Preparation, photocatalytic wettability conversion, and superhydrophobic–superhydrophilic patterning. J. Phys. Chem. C.

[11] Zubkov T, Stahl D, Thompson TL, Panayotov D, Diwald O, Yates YT (2005). Ultraviolet light-induced hydrophilicity effect on TiO_2_(110) (1*1). Dominant role of the photooxidation of adsorbed hydrocarbons causing wetting by water droplets. Phys. Chem. B.

[12] Kobayashi T, Konishi S (2015). TiO_2_ patterns with wide photo-induced wettability change by combination of reactive sputtering process and surface modification in a microfluidic channel. J. Micromech. Microeng..

[13] Maeda H, Kobayashi T, Konishi S (2017). Patterning of wettability using the photocatalytic decomposition of hydrophobic self-assembled monolayer on the TiO_2_ pattern. Jpn. J. Appl. Phys..

[14] Cassie ABD, Baxter S (1944). Wettability of porous surfaces. Trans. Faraday Soc..

[15] Wenzel RN (1936). Resistance of solid surfaces to wetting by water. Ind. Eng. Chem..

[16] Geyer FL, Ueda E, Liebel U, Grau N, Levkin PA (2011). Superhydrophobic–superhydrophilic micropatterning: Towards genome-on-a-chip cell microarrays. Angew. Chem. Int. Ed..

[17] Popova AA, Schillo SM, Demir K, Ueda E, Nesterov-Mueller A, Levkin PA (2015). Droplet-array (DA) sandwich chip: A versatile platform for high-throughput cell screening based on superhydrophobic–superhydrophilic micropatterning. Adv. Mater..

[18] Feng W, Li L, Du X, Welle A, Levkin PA (2016). Single-step fabrication of high-density microdroplet arrays of low-surface-tension liquids. Adv. Mater..

[19] Benz M, Molla MR, Böser A, Rosenfeld A, Levkin PA (2019). Marrying chemistry with biology by combining on-chip solution-based combinatorial synthesis and cellular screening. Nat. Commun..

[20] Tung YC, Hsiao AY, Allen AG, Torisawa Y, Ho M, Takayama S (2011). High-throughput 3D spheroid culture and drug testing using a 384 hanging-drop array. Analyst.

[21] Hsiao AY (2012). Micro-ring structures stabilize microdroplets to enable long term spheroid culture in 384 hanging-drop array plates. Biomed. Microdevice.

[22] Frey O, Misun PM, Fluri DA, Hengstler JG, Hierlemann A (2014). Reconfigurable microfluidic hanging-drop network for multi-tissue interaction and analysis. Nat. Commun..

[23] Birchler A (2015). Seamless combination of fluorescence-activated cell sorting and hanging-drop networks for individual handling and culturing of stem cells and microtissue spheroids. Anal. Chem..

[24] Zhang P (2016). High-throughput superhydrophobic microwell arrays for investigating multifactorial stem cell niches. Lab Chip.

[25] Maeda, H., Ohya, C., Kobayashi, T. & Konishi, S. Contact fusion of droplets patterned on opposing plates for cellular transportation and medium exchange for hanging-droplet cell culture. *Proc. Int. Conf. of Transducers*, 115–118 (2017).

[26] Ohya, C. & Konishi, S. Droplet height control by electrowetting-on-dielectric for selective contact fusion of droplets on facing substrates. *Proc. IEEE Int. Conf. of MEMS*, 1201–1204 (2018).

[27] Nelson WC, Kim CJ (2012). Droplet actuation by electrowetting-on-dielectric (EWOD): A review. J. Adhes. Sci. Technol..

[28] Wang, T. T., Huang, P. W. & Fan, S. K. Droplets oscillation and continuous pumping by asymmetric electrowetting. *Proc. IEEE Int. Conf. of MEMS*, 174–177 (2006).

[29] Koo B, Kim CJ (2013). Evaluation of repeated electrowetting on three different fluoropolymer top coatings. J. Micromech. Microeng..

[30] Hao C (2014). Electrowetting on liquid-infused film (EWOLF): Complete reversibility and controlled droplet oscillation suppression for fast optical imaging. Sci. Rep..

[31] Bormashenko E (2015). Progress in low voltage reversible electrowetting with lubricated polymer honeycomb substrates. RSC Adv..

